# Effect of Electromyography Findings of Traction‐Related Signal Loss on Prognosis During Neuromonitored Thyroidectomy

**DOI:** 10.1002/hed.70332

**Published:** 2026-05-24

**Authors:** Serkan Karaisli, Selda Gucek Haciyanli, Emine Ozlem Gur, Mehmet Haciyanli

**Affiliations:** ^1^ Department of General Surgery Izmir Kâtip Celebi University Ataturk Training and Research Hospital Izmir Turkey

**Keywords:** intraoperative neuromonitoring, loss of signal, thyroidectomy, traction injury, vocal cord paralysis

## Abstract

**Background:**

Intraoperative neuromonitoring (IONM) is a reliable tool to detect electromyographic loss of signal (LOS). Here, we investigated the effect of electromyography features of traction‐related LOS on prognosis.

**Methods:**

Thyroidectomy patients with a traction‐related LOS were included. The cases were divided into four groups as follows: Type 1 LOS with no amplitude (0 μV) (Group 1), Type 1 LOS with low‐amplitude (1–99 μV) (Group 2), Type 2 LOS with no amplitude (Group 3), and Type 2 LOS with low‐amplitude (Group 4).

**Results:**

A total of 788 patients (1429 nerves) were analyzed. Traction‐related LOS was found in 74 patients (9.4%) and 77 nerves (5.4%). Intraoperative recovery was observed in 21 (48.8%) nerves in Group 4, and significantly higher (*p* = 0.011). Vocal cord paralysis was less in Group 4 (*p* = 0.001).

**Conclusions:**

In traction‐related injury, electromyography features may contribute to the decision‐making process. Type 2 LOS with low‐amplitude is associated with a high likelihood of intraoperative recovery, potentially reducing the need for staged thyroidectomy.

## 1 Introduction

Thyroidectomy is the most commonly performed endocrine gland surgery. Recurrent laryngeal nerve (RLN) injury is a relatively frequent and devastating complication of thyroid surgery, which is a major cause of surgical malpractice claims [1].

During the identification and dissection of the RLN in thyroidectomy, several surgical maneuvers such as traction, electrocoagulation, clamping, instrumental trauma, and transection may cause RLN injury. The most common cause is traction, accounting for 70%–80% of cases, followed by thermal injury. Other rare causes include compression, clamping, ligature, aspiration, and transection [1, 2, 3].

In the past, RLN integrity and possible injury could only be assessed visually. Since visible trauma could not be found in the majority of traction‐related injuries, detecting traction‐related RLN injury intraoperatively was highly challenging [1]. Intraoperative neuromonitoring (IONM) is a widely accepted and reliable tool to detect electromyographic loss of signal (LOS), which indicates RLN injury, even if the anatomical integrity of the nerve is preserved during thyroid surgery [2, 4, 5, 6, 7].

An unchanged electromyography amplitude after nerve dissection ensures a normal vocal cord function postoperatively. However, a decreased amplitude foreshadows a functional deficiency of RLN. Complete LOS after nerve dissection may show complete loss of nerve conduction and postoperative vocal cord paralysis (VCP). However, the relationship between the decrease in partial amplitude on electromyography and the sufficient muscle strength required for normal vocal cord mobility remains under debate [8].

Intraoperative detection of LOS is important for predicting postoperative VCP. Its contribution in revising operative approach such as preference for staged thyroidectomy to prevent bilateral VCP [9]. When LOS is detected without any sign of mechanical trauma on the RLN, the first step is to determine the presence of any injury point by stimulating the RLN from the laryngeal entry point to the vagus nerve. LOS may occur due to segmental (Type 1) or global (Type 2). Type 1 LOS is defined as LOS only at the proximal segment of a specific nerve injury point. Type 2 LOS is characterized by complete unresponsiveness to the stimulation of the RLN and vagus nerve [6, 7]. Type 1 LOS has been considered to be associated with a worse prognosis [2, 3, 6, 7]. The intraoperative and postoperative recovery rates are reported to be significantly lower for patients with Type 1 LOS than for patients with Type 2 LOS [6]. Traction‐related LOS has been reported to be seen in 8.6%–11.7% of the patients and in 3.9%–7% of nerves at risk during neuromonitored thyroidectomy [2, 3, 6].

Although some studies have investigated prognosis following Type 1 and Type 2 traction‐related LOS, few have evaluated prognosis in relation to amplitude features [2]. Here, we aimed to evaluate the effect of a combination of injury type and electromyography features of the traction‐related LOS on prognosis during thyroidectomy with intermittent IONM (i‐IONM).

## 2 Materials and Methods

Patients who underwent a thyroidectomy between January 2015 and May 2024 in the Division of Endocrine Surgery of our institute were analyzed retrospectively. Patients who underwent thyroidectomy with i‐IONM and had traction‐related LOS were included. Patients with preoperative VCP, in which a continuous IONM/dissector probe was used, and with LOS due to other causes were excluded.

All operations were performed by four high‐volume (> 50 thyroidectomies per year) endocrine surgeons under general anesthesia. In cases of malignancy or suspected malignancy, the operation began from the tumor side, whereas in benign diseases, it began from the side with the larger nodule. Dr. Langer Avalanche monitor (Avalanche XD, Waldkirch, Germany) and a disposable sterile Dr. Langer bipolar stimulator probe and endotracheal surface electrodes were used for i‐IONM. The i‐IONM was used in accordance with the criteria established by the International Neural Monitoring Study Group [10]. Preoperative indirect laryngoscopy (L1), intraoperative pre‐excision and post‐excision amplitudes of vagus nerve, RLN, and external branch of superior laryngeal nerve (V1, R1, EBSLN1, and V2, R2, EBSLN2) were obtained, and postoperative Day 1 indirect laryngoscopy (L2) was performed in all patients.

The LOS was defined as an amplitude < 100 μV with vagal stimulation with a current above the vagal threshold (1–2 mA) following an adequate baseline amplitude of vagal nerve and RLN in the dry surgical field [10]. In all cases of LOS, a standardized troubleshooting algorithm was applied in accordance with the International Neural Monitoring Study Group guidelines [11]. This included verification of endotracheal tube position, confirmation of the absence of additional doses of muscle relaxant administration, exclusion of technical problems, stimulation under a dry surgical field, and vagus nerve stimulation. Additionally, the laryngeal twitch response was routinely assessed for all patients to differentiate true LOS from false‐positive events.

Traction‐related LOS was determined after exclusion of other reasons that caused visible damage on anatomical integrity. In all patients, surgical traction was terminated, and 1 mg/kg methylprednisolone was administered intravenously at the time LOS was detected. RLN amplitude was checked every 5 min. If no intraoperative recovery of LOS occurred after 20 min of waiting without surgical maneuvers, the operative strategy was reviewed. In patients who have performed a concomitant lateral neck dissection due to lymph node involvement following thyroidectomy, affected RLN amplitude was checked every 5 min, even if 20 min of standard waiting time was ended.

According to the proximal RLN amplitude at the time that first LOS detected, the LOS cases were divided into four groups: Type 1 (segmental) LOS with no amplitude (0 μV) (Group 1), Type 1 LOS with low‐amplitude (1–99 μV) (Group 2), Type 2 (global) LOS with no amplitude (Group 3), and Type 2 LOS with low‐amplitude (Group 4).

Demographic and clinical features of the groups and recovery characteristics of RLN and VCP were evaluated. All patients were followed‐up for at least 6 months. Patients with VCP underwent indirect laryngoscopy once per month for 6 months or until recovery.

Approval for this study was received from the Health Research Ethics Committee of our institute (02/13/25‐0052).

### 2.1 Statistical Analysis

Data were evaluated using the IBM SPSS Statistics Standard Concurrent User V 26 (IBM Corp., Armonk, New York, USA) statistical package program. Descriptive statistics were reported as numbers (*n*), percentages (%), and median (minimum–maximum) values. The normal distribution of data belonging to numerical variables was evaluated using the Shapiro–Wilk normality test. If the result of the variance analysis was significant, the Dunn–Bonferroni test was used as a multiple comparison test. Pearson's and Fisher's exact tests were used to compare categorical variables. A *p* < 0.05 value was considered statistically significant.

## 3 Results

After initial evaluation, a total of 788 patients were analyzed. The nerves at risk were 1429, including 641 bilateral and 147 unilateral RLNs. Traction‐related LOS occurred in 74 patients (9.4%) and affected 77 nerves (5.4%) (Figure 1). There were 7, 14, 13, and 43 nerves with traction‐related LOS in Groups 1, 2, 3, and 4, respectively. The median age was 52 (21–86) years. About 63.6% (*n* = 49) of the patients were female. The final histopathology revealed a benign thyroid disease in 50.6% (*n* = 39) of the side in which LOS occurred. The left and first‐operated sides were more commonly affected. Gender, age, dominant nodule size, baseline V1 amplitude, presence of hyperthyroidism, presence of previous neck surgery, undergoing concomitant central lymph node dissection and parathyroidectomy, final histopathology, and LOS side were comparable between groups (*p* > 0.05) (Table 1). The four high‐volume endocrine surgeons who performed all thyroidectomies in the study group had similar LOS incidence (*p* > 0.05).

**FIGURE 1:**
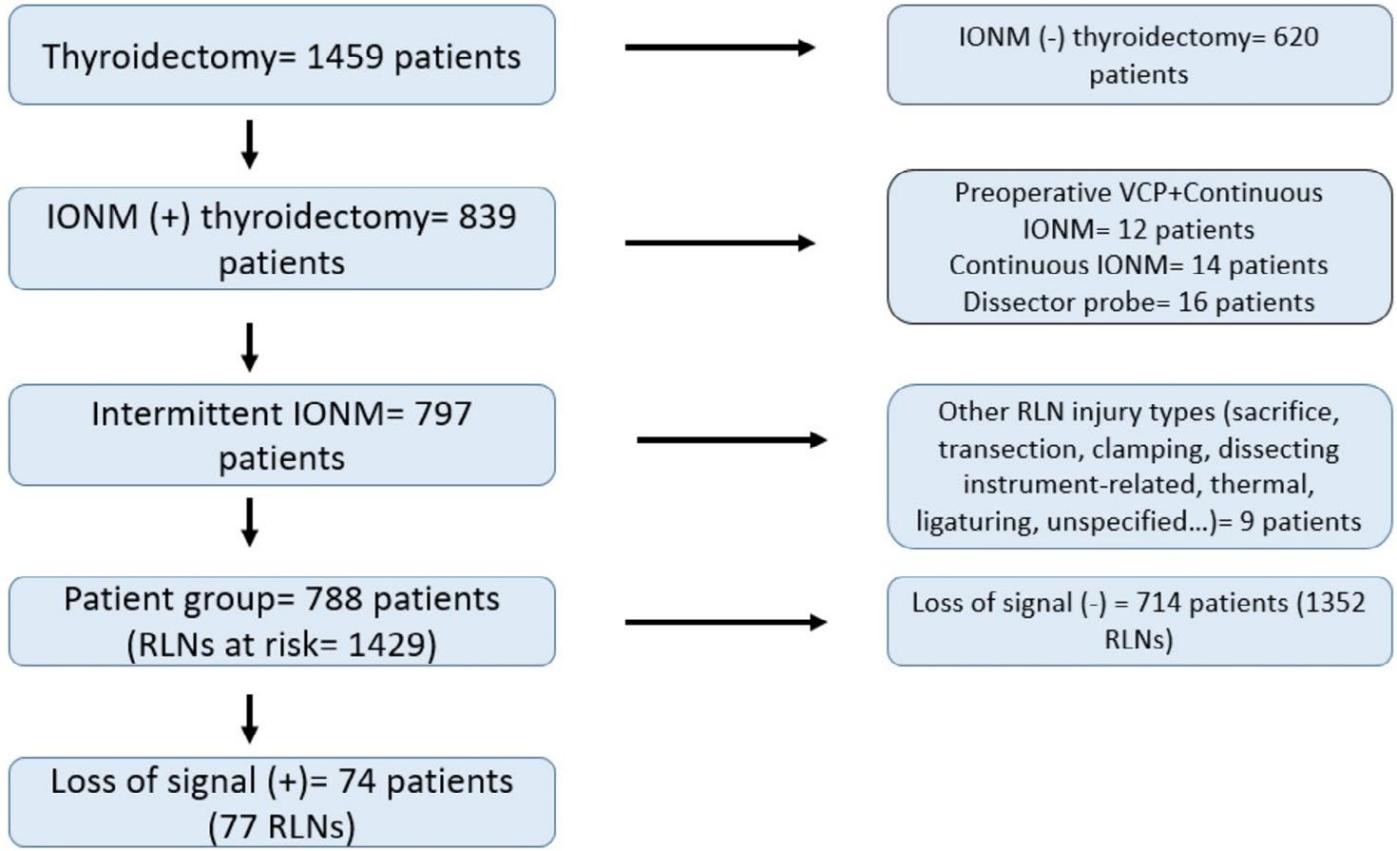
Flowchart of the study for the inclusion and exclusion of the patients. IONM, intraoperative neuromonitoring; RLN, recurrent laryngeal nerve; VCP, vocal cord paralysis. [Color figure can be viewed at wileyonlinelibrary.com]

**TABLE 1:** Comparison of a (*n* = Affected nerves).

		Group 1 (*n* = 7)	Group 2 (*n* = 14)	Group 3 (*n* = 13)	Group 4 (*n* = 43)	Total (*n* = 77)	*p*
Gender	Male	4 (57.1%)	5 (35.7%)	6 (46.2%)	13 (30.2%)	28 (36.4%)	0.438^c^
	Female	3 (42.9%)	9 (64.3%)	7 (53.8%)	30 (69.8%)	49 (63.6%)	
Age (years)	61 (46–71)		42.5 (26–67)	52 (32–77)	51 (21–86)	52 (21–86)	0.171^a^
Dominant nodule size (mm)	35 (0–55)		12 (0–50)	22 (8–85)	20 (0–125)	20 (0–125)	0.277^b^
Baseline V1 amplitude	203 (105–310)		197 (106–420)	210 (104–310)	215 (112–430)	210 (104–430)	0.644^b^
Hyperthyroidism	Yes	1 (14.3%)	6 (42.9%)	2 (15.4%)	9 (20.9%)	18 (23.4%)	0.323^c^
	No	6 (85.7%)	8 (57.1%)	11 (84.6%)	34 (79.1%)	59 (76.6%)	
Previous neck surgery	Yes	2 (28.6%)	0 (0%)	0 (0%)	5 (11.6%)	7 (9.1%)	0.083^c^
	No	5 (71.4%)	14 (100%)	13 (100%)	38 (88.4%)	70 (90.9%)	
Central lymph node dissection	Yes	0 (0%)	3 (21.4%)	3 (23.1%)	7 (16.3%)	13 (16.9%)	0.642^c^
	No	7 (100%)	11 (78.6%)	10 (76.9%)	36 (83.7%)	64 (83.1%)	
Parathyroidectomy	Yes	0 (0%)	0 (0%)	1 (7.7%)	4 (9.3%)	5 (6.5%)	0.799^c^
	No	7 (100%)	14 (100%)	12 (92.3%)	39 (90.7%)	72 (93.5%)	
Final pathology	Benign	3 (42.9%)	6 (42.9%)	6 (46.2%)	24 (55.8%)	39 (50.6%)	0.810^c^
	Malignant	4 (57.1%)	8 (57.1%)	7 (53.8%)	19 (44.2%)	38 (49.4%)	
Side	Right	3 (42.9%)	6 (42.9%)	5 (38.5%)	18 (41.9%)	32 (41.6%)	0.999^c^
	Left	4 (57.1%)	8 (57.1%)	8 (61.5%)	25 (58.1%)	45 (58.4%)	
First/second side	First‐side	5 (71.4%)	12 (85.7%)	13 (100%)	30 (69.8%)	60 (77.9%)	0.074^c^
	Second‐side	2 (28.6%)	2 (14.3%)	0 (0%)	13 (30.2%)	17 (22.1%)	

*Note:* Numerical variables are given as median (range).

^a^ One‐way ANOVA.

^b^ Kruskal–Wallis test.

^c^ Fisher's exact test.

Intraoperative recovery of LOS occurred in 21 nerves (48.8%) in Group 4, significantly higher than in other groups (*p* = 0.011). Postoperative VCP was significantly less in Group 4 compared to other groups (*p* = 0.001) (Table 2). The rate of intraoperative recovery of LOS ranged from 32.6% to 37.5% across four endocrine surgeons. There was no statistically significant difference between surgeons regarding the rate of intraoperative recovery of LOS (*p* = 0.992). All patients with intraoperative recovery of LOS had normal vocal cord functions on the first postoperative day. The median duration of intraoperative recovery of LOS was 15 (5–30) min and similar between groups (*p* > 0.05) (Table 3). Two patients experienced a recovery of LOS 25 and 30 min later during lateral neck dissection following thyroidectomy.

**TABLE 2:** Intraoperative recovery of LOS and postoperative VCP status.

		Group 1 (*n* = 7)	Group 2 (*n* = 14)	Group 3 (*n* = 13)	Group 4 (*n* = 43)	Total (*n* = 77)	*p*
Intraoperative recovery of LOS	Yes	0 (0%)	2 (14.3%)	3 (23.1%)	21 (48.8%)	26 (33.8%)	**0.011** ^a^
	No	7 (100%)	12 (85.7%)	10 (76.9%)	22 (51.2%)	51 (66.2%)	
Postoperative VCP	Yes	7 (100%)	9 (64.3%)	8 (61.5%)	13 (30.2%)	37 (48.1%)	**0.001** ^a^
	No	0 (0%)	5 (35.7%)	5 (38.5%)	30 (69.8%)	40 (51.9%)	

*Note:* Bold: Statistically significant.

Abbreviations: LOS, loss of signal; VCP, vocal cord paralysis.

^a^ Fisher's exact test.

**TABLE 3:** Duration of intraoperative recovery of LOS.

	Group 2 (*n* = 2)	Group 3 (*n* = 3)	Group 4 (*n* = 21)	Total (*n* = 26)	*p*
Duration of intraoperative recovery of LOS (min)	20 (15–25)	15 (8–15)	10 (5–30)	15 (5–30)	0.409^a^

*Note:* Numerical variables are given as median (range).

Abbreviation: LOS, loss of signal.

^a^ Kruskal–Wallis test.

In three patients who had bilateral LOS, surgery was continued on the contralateral side due to intraoperative recovery on the first operated side. In these patients, LOS on the second side improved intraoperatively in one patient, but not in two patients. None of these three patients had VCP on postoperative laryngoscopy.

The presence of impaired mobility or fixed vocal cords was comparable between groups. Permanent VCP was seen in one patient each in Group 1, Group 2, and Group 3. These three patients had fixed vocal cords on the postoperative Day 1. Rates of permanent VCP were found similar between groups (*p* > 0.05) (Table 4). The median duration of recovery of VCP was 57.5 (6–210) days and was similar between groups (*p* > 0.05) (Table 5).

**TABLE 4:** Comparison of VCP features and prognosis between groups.

		Group 1 (*n* = 7)	Group 2 (*n* = 9)	Group 3 (*n* = 8)	Group 4 (*n* = 13)	Total (*n* = 37)	*p*
Postoperative vocal cord status	Impaired mobility	2 (28.6%)	2 (22.2%)	2 (25%)	7 (53.8%)	13 (35.1%)	0.440^a^
	Fixed	5 (71.4%)	7 (77.8%)	6 (75%)	6 (46.2%)	24 (64.9%)	
VCP prognosis	Temporary	6 (85.7%)	8 (88.9%)	7 (87.5%)	13 (100%)	34 (91.9%)	0.440^a^
	Permanent	1 (14.3%)	1 (11.1%)	1 (12.5%)	0 (0%)	3 (8.1%)	

Abbreviation: VCP, vocal cord paralysis.

^a^ Fisher's exact test.

**TABLE 5:** Duration of recovery of VCP.

	Group 1 (*n* = 6)	Group 2 (*n* = 8)	Group 3 (*n* = 7)	Group 4 (*n* = 13)	Total (*n* = 34)	*p*
Duration of recovery of VCP (days)	63.5 (10–80)	57 (6–210)	96 (34–192)	45 (8–154)	57.5 (6–210)	0.255^a^

*Note:* Numerical variables are given as median (range).

Abbreviation: VCP, vocal cord paralysis.

^a^ Kruskal–Wallis test.

## 4 Discussion

This study investigated the prognosis of LOS and VCP according to a combination of injury type and electromyography features of traction‐related LOS during thyroidectomy with i‐IONM. We found that patients with Type 2 LOS with low amplitude had a significantly higher rate of intraoperative LOS recovery and a lower rate of postoperative Day 1 VCP compared to other groups.

The nerve damage is related to three factors. The first is the type of injury (transection, instrumental, thermal injury, or traction, etc.). The other two factors are the strength of damage and the duration of injury. For example, for the occurrence of the traction‐related injury, a mean of a few minutes is required [6]. An international and multicenter study conducted with patients administered continuous IONM revealed that Type 1 LOS occurs quickly after RLN trauma (mean 4 s), while Type 2 LOS presents delayed in a few minutes (mean 104 s) [12]. The main advantage of continuous IONM is providing real‐time functional monitoring throughout the RLN. However, i‐IONM only provides information about the functionality of the RLN at the moment of stimulation, not about the results of surgical maneuvers between stimulations [7]. This condition may result in transient LOS events being missed between stimulations, leading to an underestimation of LOS incidence and affecting the accuracy of classification, particularly in cases of transient or rapidly reversible nerve dysfunction. We administered i‐IONM for the majority of our patients due to the higher costs associated with continuous IONM and only i‐IONM performed cases were included in this study.

Traction‐related LOS still remains a significant and relatively frequent problem during neuromonitored thyroidectomy, even in experienced hands. A study reported 44 traction‐related LOS cases, accounting for 6.8% of the 644 patients and 3.9% of the 1142 nerves at risk [3]. A study from Taiwan found a traction‐related LOS in 8.6% of the patients and in 7.0% of the nerves at risk [2]. According to another study, LOS occurred in 168 of 1442 (11.7%) patients and 171 of 2748 (6.2%) nerves at risk [6]. Recently, a lower rate of LOS (3.5%) was reported in a comprehensive study conducted with 5884 thyroidectomies [13]. Traction‐related LOS was also found to have higher rates up to 20.5% when pre‐excision and post‐excision amplitudes of vagus nerve (V1 and V2) were not routinely considered [14]. In our study, similar to previous studies, traction‐related LOS was seen in 74 (9.4%) patients and 77 (5.4%) nerves at risk.

Intraoperative recovery of LOS is observed in 70% of all traction‐related RLN injuries within 20 min after traction is terminated [2, 7]. The intraoperative recovery rate significantly depends on the type of LOS. Type 2 LOS is the most common type of traction‐related RLN injury and it has been emphasized to be associated with a better prognosis [2, 3, 6, 7]. Liu et al. [2] reported a higher intraoperative recovery rate in patients with Type 2 LOS compared to those with Type 1 LOS (74.6% vs. 28.6%). If LOS persists intraoperatively, the frequency of postoperative VCP ranges from 43.8%–95% and 14.3%–70% in patients following Type 1 and Type 2 LOS, respectively [3, 6, 7]. Similarly, in our study, Type 2 LOS was seen more frequently (72.7%), and both intraoperative recovery of LOS and detection rate of normal vocal cord examination on the first postoperative day were found to be higher compared to Type 1 LOS.

Regardless of injury type, an injury with decreased amplitude has a better prognosis than one with complete loss of electromyographic amplitude [3, 11]. An amplitude level of above 250 μV has been thought to be highly correlated with postoperative normal vocal cord function [11]. Chiu et al. [3] found a lower postoperative VCP rate in patients with a low electromyographic amplitude level, compared with patients with LOS with no amplitude (13.7% vs. 75%). We found that LOS with low amplitude had a better prognosis than LOS with no amplitude in both types of RLN injuries, which is consistent with the current literature.

From a practical perspective, the proposed classification may support intraoperative decision‐making. In cases of Type 2 LOS with low‐amplitude, where intraoperative recovery is frequent and early VCP risk is lower, continuation of surgery on the contralateral side may be considered in selective cases. In contrast, Type 1 LOS with no amplitude may warrant a more cautious approach, including consideration of staged thyroidectomy.

Dionigi et al. [1] proposed a classification of severity for RLN injury. In cases of traction‐related injuries, they observed nerve recovery in an average of 27 ± 9 days, which was the shortest recovery duration among all injury types. For this reason, traction‐related injury was classified as a mild severity injury. Another study found a duration of recovery 35.8 ± 19.4 days on average for traction‐related VCP [15]. Liu et al. [2] reported a mean VCP recovery duration of 24.4 days, with a maximum of 12 weeks, which was revealed longer in Type 1 LOS, compared to Type 2 LOS (44.8 vs. 17.6 days). Approximately 90%–100% of all these RLN injuries recover spontaneously within 6 months [2, 3, 6]. The median duration of the VCP recovery was 57.5 (6–210) days in our study, which was relatively longer than the current literature. Due to the lockdown during the pandemic period, there may have been a delay in routine patient follow‐up; consequently, the detection of VCP recovery may have taken much longer than it actually was.

The traction trauma often results in transient VCP. The rate of permanent VCP is 1.4% for traction‐related injuries, 28% for thermal, 50% for clamping, and 100% for transection injuries [1]. Permanent VCP was seen in three patients, and the rate of permanent VCP was found to be 3.9% among the patients with traction‐related LOS in our study. A relatively higher rate of permanent VCP in our study group may be due to the apparent effect of individual cases in a relatively small sample size.

This study has some limitations. Firstly, this study has a retrospective design and was conducted in a single tertiary center. Also, the presence of a small sample size may have resulted in a reduction in the power of the statistical analysis. Additionally, the study data are dependent on the accuracy of medical records. We cannot be sure about the certain reason for the injury type, although only four high‐volume endocrine surgeons prospectively recorded clinical data. Also, due to the lockdown during the pandemic period, the detection of VCP recovery may have taken much longer than it actually was. Finally, only the amplitude values when LOS first detected were considered in this study and intraoperative partial amplitude recovery was not taken into account, which prevents the evaluation of the impact of partial amplitude recovery on VCP function. Emerging evidence suggests that dynamic recovery patterns may provide additional prognostic value [13]; future prospective studies should evaluate the impact of amplitude recovery kinetics on functional outcomes. On the other hand, to the best of our knowledge, this is the first study that investigated the prognosis by considering the type of RLN injury (segmental and global) and amplitude characteristics together in the current literature. Unlike previous studies that evaluated LOS type or amplitude independently, this study is the first to integrate both parameters into a combined classification system, demonstrating that prognostic stratification improves when electrophysiological severity is interpreted in conjunction with injury pattern.

In conclusion, approximately 10% of the patients still have a risk of traction‐related LOS during neuromonitored thyroidectomy even in high‐volume endocrine surgery centers. In traction‐related RLN injury, consideration of LOS type, along with electromyographic characteristics, may predict perioperative and postoperative prognosis and guide surgical strategy. Type 2 LOS with low amplitude is the most common type of LOS and is associated with the most favorable prognosis. In Type 2 LOS with low amplitude, since approximately half of LOS cases recover intraoperatively and early postoperative VCP risk is low, the need for staged thyroidectomy can be reduced, and completion surgery can be scheduled in the early postoperative period.

## Author Contributions

Conceptualization: Serkan Karaisli, Selda Gucek Haciyanli, Mehmet Haciyanli. Methodology: Serkan Karaisli, Emine Ozlem Gur. Formal analysis and investigation: Selda Gucek Haciyanli, Emine Ozlem Gur. Writing – original draft preparation: Serkan Karaisli, Emine Ozlem Gur. Writing – review and editing: Serkan Karaisli, Emine Ozlem Gur, Mehmet Haciyanli. Funding acquisition: Selda Gucek Haciyanli, Mehmet Haciyanli. Resources: Serkan Karaisli, Selda Gucek Haciyanli, Mehmet Haciyanli. Supervision: Emine Ozlem Gur, Mehmet Haciyanli.

## Funding

The authors have nothing to report.

## Disclosure

This study was presented and awarded third place of best oral presentations at the 12th National Congress of Endocrine Surgery in Antalya‐Turkey, February 13–16, 2025.

## Ethics Statement

Ethics committee approval was obtained from the Health Research Ethics Committee of Izmir Kâtip Celebi University (Decision number: 0052, Decision date: February 13, 2025). Authors declared that the research was conducted according to the principles of the World Medical Association Declaration of Helsinki “Ethical Principles for Medical Research Involving Human Subjects,” (amended in October 2024).

## Consent

Written informed consents were obtained from the patients/patient relatives who participated in this study for this study.

## Conflicts of Interest

The authors declare no conflicts of interest.

## Data Availability

The data are available from the corresponding author on reasonable request.
